# **Female sexual function in different phenotypes of polycystic ovarian syndrome**: **a comparative cross-sectional study**

**DOI:** 10.1038/s41598-022-24026-7

**Published:** 2022-11-11

**Authors:** Mahsa Yarjanli, Shahideh Jahanian Sadatmahalleh, Negin Mirzaei, Khadijeh Azarbajani

**Affiliations:** 1 Department of Reproductive Health and Midwifery, Faculty of Medical Sciences, Tarbiat Modares University, Jalal Al-Ahmad Highway, Nasr Bridge, Tehran, 14115-111 Iran; 2 Department of medicine, School of public health, University of Adelaide, Adelaide, Australia

**Keywords:** Endocrinology, Medical research

## Abstract

Polycystic ovary syndrome (PCOS) coexisting mood disorders along with a combination of aesthetic manifestations may have a detrimental effect on women's sexual function. Hence, different phenotypes of PCOS have different clinical and biochemical signs and symptoms. The aim of this study was to compare women's sexual function (SF) in different phenotypes of PCOS. This cross-sectional study was conducted on 364 women who met the Rotterdam diagnostic criteria to compare different PCOS phenotypes (A = 95, B = 79, C = 95, and D = 95) and 100 non PCOS women in control group. All participants were invited to fill out the female sexual function index (FSFI). Significant differences were observed between the different phenotypes and control group in terms of the total score, sexual desire, arousal, lubrication, and satisfaction (*P* < 0.001); however, no significant differences were found between different phenotypes in terms of pain (*P* > 0.05) and orgasm (*P* > 0.05) but difference was significant between different phenotypes and control group. In addition, phenotype B had the lowest mean score of total FSFI (*P* < 0.05). The results indicated that women's SF is significantly different in different PCOS phenotypes. It is concluded that in order to solve the SF problems of women with PCOS, different treatment and care measures should be considered according to the relevant phenotype**.**

## Introduction

Polycystic Ovary Syndrome (PCOS) is a heterogeneous disorder that influences 6–10% of reproductive-aged women^1^. It is associated with a combination of distressing manifestations such as subfertility, irregular menstruation, acne, obesity, alopecia, and hirsutism, which may affect feminine identity, self-esteem, and body image destructively^2,3^. These aesthetic factors together with sex hormone imbalances (hyperinsulinemia, hyperandrogenism, and increased luteinizing hormone level) can trigger anxiety, depression, and worsened Quality of Life (QoL) in women^4–6^. In addition, these mood disorders and their medications can pose detrimental effects on Sexual Function (SF)^7,8^.

Four different phenotype categories have been identified for PCOS based on the National Institute of Health (NIH) consensus panel: phenotype A including Hyperandrogenism clinical or biochemical (HA), Ovulatory Dysfunction (OD), and Polycystic Ovary (PCO), Phenotypes B and C: HA + OD, and HA + PCO, respectively, and phenotype D including OD and PCO.

Furthermore, the clinical manifestations as well as metabolic and hormonal profiles are different in these phenotypes^9^. So, SF can be influenced differently in different phenotypes of PCOS.

Although clinical signs of PCOS can be deleterious for SF, the association of PCOS with SF remains inconsistent. In addition, there are limited studies specifically evaluating SF regarding different PCOS phenotypes. Therefore, the present study was conducted to compare SF of different phenotypes of PCOS and non PCOS women in Iran.

## Materials and methods

This cross-sectional study was conducted on a group of PCOS women and a healthy control group who were referred to the gynecological clinics of hospitals in Tehran province (Iran) between May 2018 and February 2019 through the convenience sampling method. The Ethics Committee of Tarbiat Modares University of Medical Sciences approved the study protocol (IR.MODARES.REC. 1397.211).

Firstly, the sample size was calculated using the appropriate formula and considering the 95% confidence interval. Consequently, 364 women with PCOS diagnosis according to the Rotterdam criteria (95 women with phenotype A, 79 women with phenotype B, 95 women with phenotype c, and 95 women with phenotype D and 100 healthy women in control group) were recruited after obtaining written consent. Eligibility criteria required for selecting the subjects were as follows: married women of reproductive age (18–45 years) who lived with a husband and were sexually active (had sexual intercourse in the past four weeks). In addition, PCOS was diagnosed based on the Rotterdam criteria and willing to participate in this study. The possible confounding factors were avoided by the exclusion criteria: pregnancy, breastfeeding, suffering from endocrine and chronic diseases (like diabetes, cardiovascular diseases, kidney disease, benign and malignant tumors, etc.), taking any hormonal and herbal medicines in the last month due to their possible impact on SF and androgen levels. The participants were asked to fill out the questionnaires, which included a series of questions about demographic characteristics and sexual dysfunction.

### Phenotypical features

Anthropometric measurements including weight, height, hip circumference (HC), and waist circumference (WC) were measured by the same person for all participants. Body mass index (BMI) was calculated based on dividing weight in kilograms by the square of height in meters for assessing obesity^10^. Additionally, WC was measured at the narrowest point between the lower rib and iliac crest in the standing position and HC was calculated at the widest part of the buttocks, dividing WC by the HC was considered the Waist to Hip Ratio (WHR)^11^.

### Clinical and para-clinical features

Clinical features such as hirsutism, menstrual cycle status, and acne were assessed by a clinician. Menstrual cycle lengths shorter than 24 days and longer than 34 days were considered abnormal. The modified Ferriman–Gallwey (mFG) was used to identify hirsutism. It consists of observing the quantity and distribution of terminal hair in nine body areas, including the upper lip, chin, chest, upper and lower abdomen, back, sacroiliac region, thighs, and arms. These areas were given a score ranging from 0 to 4 according to quantity and density, with higher scores indicating a greater amount of body hair^12^. The reliability and validity of this questionnaire have been confirmed in Iran^13^.

The degree of severity of acne was examined based on the Conference on Acne Classification, which is divided into mild, moderate, and severe. The mild form consists of small comedowns’ number of papules and nodules, with no cysts and scars. Although there is a very large number of papules and posture in the medium form of acne, nodules, cysts, and scars are seen rarely. In the severe form, the number of papules and pustules is very large and the number of nodules, cysts, and scars is also high^14^.

Polycystic ovary morphology (PCOM) was detected based on the Rotterdam PCOS criteria: An ovarian volume > 10 mL or containing 12 or more follicles (2–9 mm) in size was distinguished as a positive PCOM.

All blood samples which contained LH, FSH, Testosterone, SHBG, TSH, progesterone and prolactin were measured on day-3 of the menstrual cycle by the same laboratory tests (ELISA method). Also, the Free Androgen Index (FAI) value was calculated by dividing the total testosterone (nmol/lit)/SHBG multiplied × 100.

### Questionnaire

A socio-demographic questionnaire (including age, marital status, employment status, educational level, BMI, history of chronic disease, menstrual and reproductive history such as duration and length of the menstrual cycle, regularity of cycle, number of children, and abortion) was completed.

### Sexual function

To assess SF, all participants were asked to fill out the Female Sexual Function Index (FSFI), which includes 19 questions to measure women's SF in six areas: desire, arousal, moisture, orgasm, satisfaction, and pain. The evaluation was done through the Likert scale. The total score was obtained by summing the six domain scores. A higher score is associated with a lower degree of sexual dysfunction and the total score of 26.55 is the optimal cut score for differentiating women with and without sexual dysfunction^15^. The reliability and validity of this questionnaire have been confirmed in Iran^16^.

### Statistical method

Statistical analysis was performed by using the SPSS software (Version 16, SPSS Inc, Chicago, IL, USA). The normality of data was assessed using Kolmogorov–Smirnov's test and presented as mean + SD for normal and quantitative data. A one-way ANOVA was used with Bonferroni Post Hoc test to compare normal variables between the groups, and Kruskal–Wallis test was used to compare non-normal variables between different groups. Also, to compare qualitative variables between the different groups, Chi-square test, Fisher's exact test, Fisher's generalized test were used. *P* values lower than 0.05 were considered statistically significant.

### Ethics approval and consent to participate

This study was approved by the Institutional Review Board, and the Ethics Committee of Tarbiat Modares University of Medical Sciences approved the study protocol. All procedures were in accordance with the ethical standards of the Regional Research Committee, as well as the Declaration of Helsinki 1964 and its later amendments. After explaining the study's purposes, informed written consent and verbal assent was obtained from all participants. They were informed that their participation was voluntary, confidential, and anonymous and that they had the right to withdraw from the research at any time.

## Results

**Figure 1:**
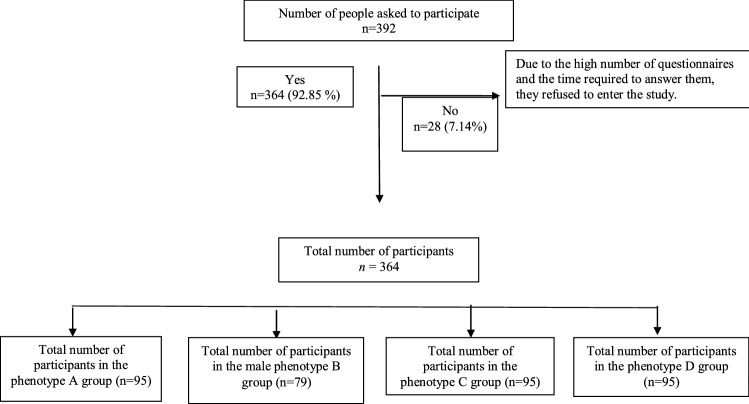
Flow chart of the study.

Among the 492 participants interviewed for this study, 28 women didn’t meet the inclusion criteria (7%) (Fig. 1). Table 1 illustrates some of the basic features of PCOS diagnosis in each group.

**Table 1:** Basic features of PCOS diagnosis in each phenotype group.

Variable	HA + PCOM + OD (A) n = 95	HA + OD (B) n = 79	HA + PCOM (C) n = 95	OD + PCOM (D) n = 95	Normal range
TT *	2.67 ± 1.99	1.92 ± 1.42	1.71 ± 1.46	1.18 ± 0.49	0.3–1.23 **
FAI*	8.90 ± 7.70	5.51 ± 3.58	4.01 ± 4.49	3.00 ± 2.91	1–5.1**
SHBG *	41.49 ± 27.20	39.82 ± 20.19	51.10 ± 28.29	50.71 ± 23.00	41.8–125.2 ***

*PCOS* Polycystic Ovary Syndrome, *PCOM*: Polycystic Ovaries Morphology, *OD* Ovulatory Dysfunction, *HA* Hyperandrogenism, *TT* Total Testosterone, *SHBG* Sex Hormone Binding Globulin, *FAI* Free Androgen Index.

*Data are given as Mean ± SD. ** ng/dL, *** nmol/L.

Table 2 compares the demographic characteristics of different phenotypes of PCOS and control group. As can be seen, there are no significant differences in the women’s age, BMI, WC, HC, WHR, education level, occupation status, number of children, number of abortions and fertility status between group.

**Table 2:** Comparison of demographic and anthropometric characteristics between different PCOS phenotypes and control group.

Variable	HA + PCOM + OD (A) N = 95	HA + OD (B) N = 79	HA + PCOM (C) N = 95	OD + PCOM (D) N = 95	Control N = 100	*P* value^¥^
Age*	29.62 ± 5.44	31.32 ± 4.84	30.95 ± 5.13	31.18 ± 5.28	29.95 ± 4.10	0.12
BMI*	27.05 ± 4.40	26.01 ± 31.55	25.96 ± 3.99	25.87 ± 3.59	26.98 ± 4032	0.09
WC**	47.79 ± 42.50	39.64 ± 42.72	47.57 ± 42.14	47.34 ± 41.14	48.34 ± 42.06	0.94
HC**	55.46 ± 48.07	45.71 ± 47.95	54.33 ± 48.05	52.70 ± 46.97	55.11 ± 47.88	0.54
WHR**	0.85 ± .012	0.86 ± 0.11	0.85 ± 0.04	2.06 ± 10.16	0.86 ± 0.04	0.13
Education level***						
≤ 12	58 (61.1)	33 (41.8)	33 (41.8)	45 (47.4)	47 (47)	0.15
> 12	37 (38.9)	36 (45.6)	44 (46.3)	44 (46.3)	53 (53)	
Occupation status***						
Housewife	82 (86.3)	66 (83.5)	78 (82.1)	71 (74.7)	72 (72)	0.07
Employed	13 (13.7)	13 (16.5)	17 (17.9)	24 (25.3)	28 (28)	
Children number*						
0	58 (61.1)	28 (35.4)	64 (67.4)	55 (57.9)	31 (31)	0.14
1	27 (28.4)	39 (49.4)3	22 (23.2)	31 (32.6)	55 (55)	
2 ≤	10 (10.6)	12 (15.2)	9 (9.5)	9 (9.5)	14 (14)	
Abortion number*						
**0**	76 (80)	59 (74.7)	78 (82.1)	69 (72.6)	79 (79)	0.51
**1**	10 (10.5)	14 (17.7)	10 (10.5)	16 (16.8)	10 (10)	
**2 ≤ **	9 (9.5)	6 (7.6)	7 (7.4)	10 (10.6)	11 (11)	
Infertility***						0.86
**Yes**	4 (4.2)	5 (6.3)	4 (4.2)	5 (5.3)	3 (3)	
**No**	91 (95.8)	74 (93.7)	91 (95.8)	90 (94.7)	97 (97)	

*PCOS* Polycystic Ovary Syndrome, *PCOM* Polycystic Ovaries Morphology, *OD* Ovulatory Dysfunction, *HA* Hyperandrogenism, *WC* Waist Circumference, *BMI* Body Mass Index, *WHR* Waist to Hip Ratio, *HC* Hip Circumference.

*Values are given as Mean ± SD using the ANOVA test.

** Values are given as Mean ± SD using the Kruskal–Wallis test.

***Values are given as number/percent using the Chi-square test.

¥ Pairwise comparison with level 1%.

Table 3 presents the summary statistics for the comparison of FSFI characteristics between different groups. As shown, there is a significant difference between the PCOS categories and control group in the mean total score of FSFI, sexual desire, arousal, lubrication, satisfaction, and orgasm (*P* < 0.05). However, no significant differences were found between these groups in terms of pain (*P* > 0.05). In addition, there are significant differences between the scores of phenotype B and other categories in all SF sub-groups except orgasm (*P* < 0.05).

**Table 3:** Comparison of FSFI and its domains between different phenotypes of PCOS.

Variable	HA + PCO M + OD (A) N = 95	HA + OD (B) N = 79	HA + PCOM (C) N = 95	OD + PCO M (D) N = 95	Control N = 100	*P* value*	Pair/wise comparisonP-value**
Desire	3.78 ± 0.90	3.39 ± 0.81	3.63 ± 0.76	3.70 ± 0.98	4.36 ± 0.98	< 0.001	A and B: 0.005
							A and C: 0.24
							A and D: 0.76
							B B and C: < 0.001
							B and D: < 0.001
							C and D: 0.38
							A and control < 0.001
							B and control < 0.001
							C and control < 0.001
							D and control < 0.001
Arousal	4.05 ± 1.08	3.06 ± 1.04	4.02 ± 1.06	3.99 ± 1.03	5.85 ± 0.38	< 0.001	A and B: < 0.001
							A and C: 0.72
							A and D: 0.82
							B and C: < 0.001
							B and D: < 0.001
							C and D: 0.88
							A and control < 0.001
							B and control < 0.001
							C and control < 0.001
							D and control < 0.001
Lubrication	4.51 ± 1.01	4.08 ± 3.74	4.93 ± 0.98	4.56 ± 0.98	5.41 ± 0.77	< 0.001	A and B: < 0.001
							A and C: 0.001
							A and D: 0.909
							B and C: < 0.001
							B and D: 0.003
							C and D: 0.003
							A and control < 0.001
							B and control < 0.001
							C and control < 0.001
							D and control < 0.001
Orgasm	4.56 ± 1.13	4.15 ± 1.10	4.52 ± 1.11	4.58 ± 1.05	5.26 ± 0.57	< 0.001	A and B: .240
							A and C: 1.00
							A and D: 1.00
							B and C: .190
							B and D: .831
							C and D: 1.00
							A and control < 0.001
							B and control < 0.001
							C and control < 0.001
							D and control < 0.001
Satisfaction	4.64 ± 1.14	4.25 ± 1.07	4.85 ± 1.08	4.86 ± 1.15	5.03 ± 0.79	< 0.001	A and B: 0.005
							A and C: 0.21
							A and D: 0.76
							B and C: < 0.001
							B and D:0.02
							C and D: 0.15
							A and control.256
							B and control < 0.001
							C and control 0.32
							D and control 0.41
Pain	3.41 ± 1.24	3.65 ± 1.19	3.69 ± 1.45	3.57 ± 1.30	4.85 ± 0.81	< 0.001	A and B: 1.00
							A and C: 1.00
							A and D: 1.00
							B and C: 1.00
							B and D: 1.00
							C and D: 1.00
							A and control < 0.001
							B and control < 0.001
							C and control < 0.001
							D and control < 0.001
Total FSFI	25.00 ± 4.22	18.61 ± 8.60	25.56 ± 4.63	24.77 ± 4.73	30.77 ± 1.35	< 0.001	A and B: < 0.001
							A and C: 0.34
							A and D :0.75
							B and C: < 0.001
							B and D: < 0.001
							C and D: 0.27
							A and control < 0.001
							B and control < 0.001
							C and control < 0.001
							D and control < 0.001

*PCOS* Polycystic Ovary Syndrome, *PCOM* Polycystic Ovaries Morphology, *OD* Ovulatory Dysfunction, *HA* Hyperandrogenism, *FSFI* Female Sexual Function Index.

*Values are given as Mean ± SD by using Kruskal–Wallis test.

**Pairwise comparison with Bonferroni test level 1%.

## Discussion

In this study, for the first time, SF was compared between different confirmed PCOS phenotypic categories in a representative sample of Iranian women. According to the obtained results, there were significant differences in terms of all FSFI domains except orgasm and pain between different phenotypes of PCOS. It was also found that sexual symptoms’ scores in phenotype B were significantly lower as compared with the other phenotypes. This finding is consistent with Bahadori et al.’s study, they also reported that phenotype B had the lowest mean score in the FSFI and SF-12 questionnaires. Furthermore, a significant difference was observed between the women with PCOS and the control group in terms of arousal, lubrication, pain, and mean total score of FSFI^17^.

The SF of PCOS women is affected by the interaction of many factors. Firstly, although there is a positive association between androgen levels and enhanced quality of SF, the HA manifestations can adversely affect the patients’ mental health^18^. Hirsutism, acne, and overweight often manifest when SF and raising a family is very important. These alternations in appearance and aesthetic standards allow PCOS women to report a feeling of unattractiveness and less feminine^19^, leading to psychosocial implications and decreased sexual self-worth^20^.Tian et al. reported that women with more severe clinical symptoms of hyperandrogenism had a lower sexual function score^21^. Secondly, emotional and social discomforts concomitant with long-term health risks can cause impaired SF and psychological well-being^19^. In addition, PCOS women are at an increased risk for mood disorders, such those 14 to 67% of patients who have been reported to suffer from depressive symptoms^22^. As a matter of fact, increased incidence of depression could result from frequent rate of infertility^23^; additionally, complaining of reduced libido has been reported by infertile women and those experiencing recurrent abortion^24^. In the study by Naumova et al.^25^ with the aim of comparison of psychological issues and sexual function in women with different infertility Causes, they reported that the prevalence of depression and anxiety was significantly higher in infertile women with PCOS than other infertile group. Hyperandrogenism and overweight was associated with higher incidence of depression and anxiety and the severity of anxiety symptom was associated with the status and response to infertility treatments. Finally, the results of their study showed that women's sexual performance in the areas of orgasm and satisfaction was impaired. Stapinska-Syniec et al.^26^ outlined, however, that infertility does not have isolated impact on depression in PCOS women.

Many studies have revealed that although having the same frequency of sexual intercourses and fantasies as controls, PCOS women are less satisfied and sexually attractive^27^. Stovall et al.^28^ reported that women with PCOS are comparable with the controls in SF scores except orgasm domain. Since HA is considered as a hallmark of PCOS, they evaluated the association of serum testosterone levels and SF. Overall, it is argued that minimum levels of testosterone are associated with the lowest scores of SF; surprisingly, higher testosterone levels are associated with higher desire/frequency rather than desire/interest scores^28^. Mantzou et al. demonstrated that the adverse effect of PCOS on women's sexual performance is largely dependent on the hormonal changes that caused by it and independent of BMI and ovulation disorders are considered as an important factor in determining the prognosis of PCOS on the sexual performance of affected women^29^.

In the present study, there is significant difference between control group and groups with different phenotypes of PCOS in terms of desire, arousal, lubrication, orgasm, pain and satisfaction that can confirm the hypothesis of the detrimental effects of PCOS on Sf. In addition, women with phenotype B PCOS had more impaired SF than the other phenotypes. These results can be explained by hormonal differences. In a recent study by the Lizneva et al.^30^, phenotypes A and B were considered as classic categories possessing more menstrual dysfunction, higher androgen and insulin levels, increased rate of insulin resistance, and being at higher risk of metabolic syndrome and obesity comparing to non-HA phenotypes. In addition, the highest antimüllerian hormone levels were found in the classic PCOS categories.

SF is reportedly more impaired in phenotype B rather than in other groups, possibly due to HA. Although HA has a prominent role in PCOS diagnosis^31^, the association between levels of androgen and SF remains inconsistent. Previous studies have shown that serum testosterone levels are associated positively with SF^6,32^; however, Ercan et al.^33^ demonstrated an inverse relationship. In addition, two large studies showed that there is not any association between SF and the levels of androgen (total and free testosterone and free androgen index^34,35^. Morotti et al.^36^found that lean PCOS patients have been considered the same as non-PCOS women in terms of sexual behavior, meaning that moderate hirsutism and HA do not have an important impact on their self-esteem and body image. Contrary to these results, another study in non-PCOS women supports the positive effect of high level of testosterone on the psychological experiences of orgasm in PCOS women^37^. In addition, these results are in agreement with the findings of Dilbaz et al. who analyzed the health-related QOL scores between infertile women and different PCOS phenotypes. The levels of hirsutism, primary infertility and phenotype HA-AO were correlated with QOL scores^38^.

Furthermore, irregular menstrual cycle, which characterizes PCOS, can manifest in classic phenotypes more than others^30^ Although considered as a distress factor that can impair psychological health, menstrual irregulation does not have confirmed effects on SF^39^.

According to the fact that infertility is considered as one of the main confounders of sexual function that has been omitted in this study to assess the effects of PCOS on SF. Although the main strength of this study includes evident phenotypic classification, it has some limitations such as lack of non-PCOS group to compare the results, and relatively small sample size in each group. Furthermore, the lack of psychological evaluation before recruiting the participants is considered one of the limitations of this study and the result would have been more reliable if we could assess their psychological health.

## Conclusion

The results of this study indicated significant differences in terms of SF and its domains in different phenotypes of PCOS. To treat sexual dysfunction in women with PCOS, different treatment and care measures should be considered according to the relevant phenotype. Accordingly, further studies are needed to identify and overcome this difficulty in different populations.

## Data Availability

The data sets used and analyzed for the current study are available upon reasonable request of the corresponding author Dr. Shahideh Jahanian (shahideh.jahanian@modares.ac.ir).

## Abbreviations

PCOS: Polycystic ovary syndrome
PCO: Polycystic ovaries
NIH: National institute of health
DHEAS: Dehydroepiandrosterone sulfate
OD: Ovulatory dysfunction
FAI: Free androgen index
LH: Luteinizing hormone
FSFI: Female sexual function index
FSH: Follicle stimulating hormone
WC: Waist circumference
BMI: Body mass index
WHR: Waist to hip ratio
HA: Hyperandrogenism
TT: Total testosterone
HC: Hip circumference
MW: Mann–Whitney
QoL: Quality of life
FSD: Female sexual dysfunction
mFG: Modified Ferriman–Gallwey
